# Bilateral ureteroinguinal hernia into the scrotum repaired in a staged fashion

**DOI:** 10.1093/jscr/rjac591

**Published:** 2022-12-28

**Authors:** Jason Beckermann, Drake Giese, Michael Rentzepis, Christopher Johnson, Joseph Wildenberg

**Affiliations:** General and Trauma Surgery, Mayo Clinic Health System, Eau Claire, WI 54702, USA; Medical College of Wisconsin—Central Wisconsin Wausau, WI 54401, USA; Urology, Mayo Clinic Health System, Eau Claire, WI 54703, USA; General and Trauma Surgery, Mayo Clinic Health System, Eau Claire, WI 54703, USA; Vascular and Interventional Radiology, Mayo Clinic Health System, Eau Claire, WI 54703, USA

## Abstract

Bilateral ureteroinguinal hernia is a rare presentation with only five cases previously presented in the literature. We describe a 60-year-old male who was diagnosed with large bilateral hernias extending into the scrotum containing redundant ureters. Both hernias were repaired in staged fashion with retrograde temporary ureteral stent placement and modified Lichtenstein technique. Follow-up computerized tomography imaging with >5 years follow-up revealed intact repairs with no evidence of urinary obstruction.

## INTRODUCTION

Inguinal hernia repair is one of the most common operations performed in the United States with over 800 000 surgeries annually [1]. Ureteroinguinal herniation is rare with <70 cases reported in English literature [2]. Our review of the literature identified five cases of bilateral ureteroinguinal hernia [3–7]. There is only one previous case in which bilateral repair has been reported [7].

Most patients diagnosed with herniation of urologic organs have no symptoms of urologic pathology. The most common diagnosis is in obese men in their 50s or 60s [4]. Some patients are diagnosed with urinary frequency or urgency, sepsis, hydronephrosis, obstruction and acute kidney injury. Most published cases were identified at the time of surgical exploration or post-operatively as the result of a complication [8]. It has been reported that 23.5% of patients with herniation of urologic organs are associated with complications [9].

We report a case of large bilateral ureteroinguinal scrotal hernia identified pre-operatively and repaired successfully in a staged fashion.

## CASE REPORT

**Figure 1:**
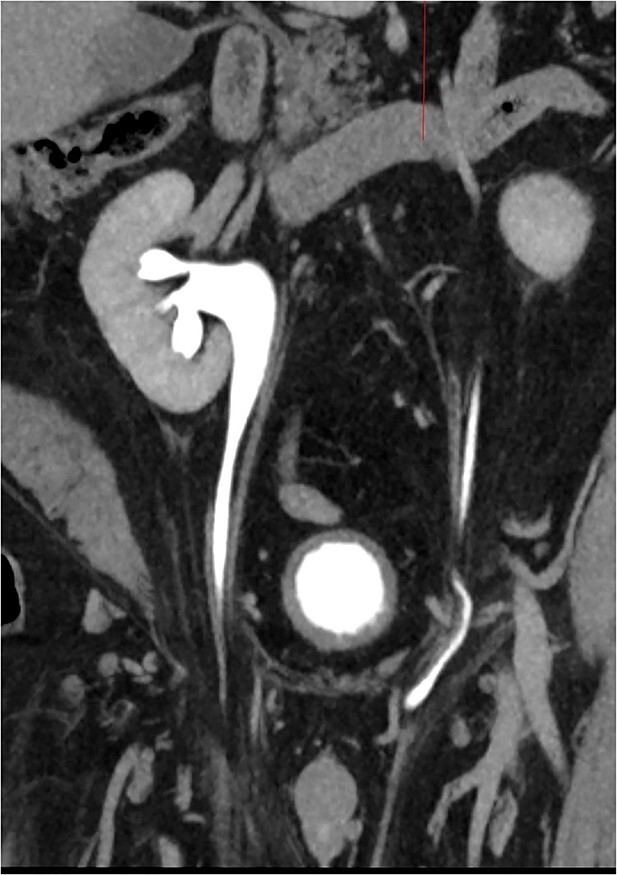
CT Urogram with bilateral ureteroinguinal scrotal hernia. Maximum intensity projection image has been reformatted to highlight both ureters crossing the inguinal canals.

This is a 60-year-old male with a body mass index of 45.4 who was referred to the emergency room with a complaint of acute onset left flank pain. Past medical history was significant for hypertension and hyperlipidemia. Past surgical history was notable for appendectomy and multiple right lower extremity operations as a child to correct deformities from polio. He continued to require right lower extremity bracing. At the time of diagnosis, he had no additional gastrointestinal or urinary symptoms. Examination was notable for bilateral groin bulge suggestive of hernia versus hydrocele. The complete blood count, basic metabolic panel and urinalysis were unremarkable. A computerized tomography (CT) scan of the abdomen was performed and revealed bilateral inguinal hernias containing ureters and fatty tissue (Fig. 1). Mild right hydronephrosis was noted. Pain improved after the administration of ketorolac and patient was referred to general surgery for further evaluation.

**Figure 2:**
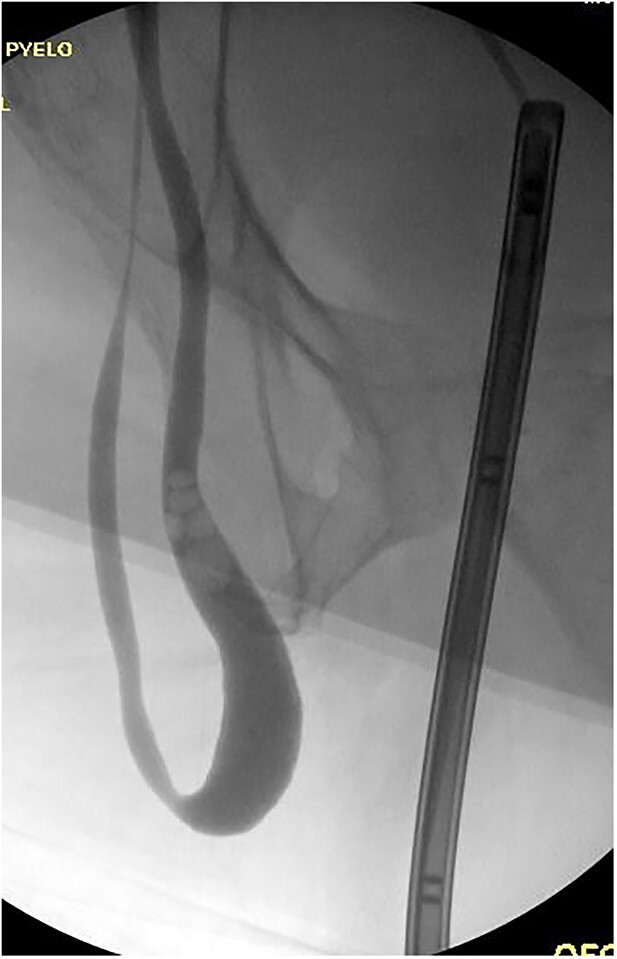
Retrograde pyelogram exhibiting ureter herniated into scrotum prior to hernia repair. Filling defects consistent with air bubbles.

**Figure 3:**
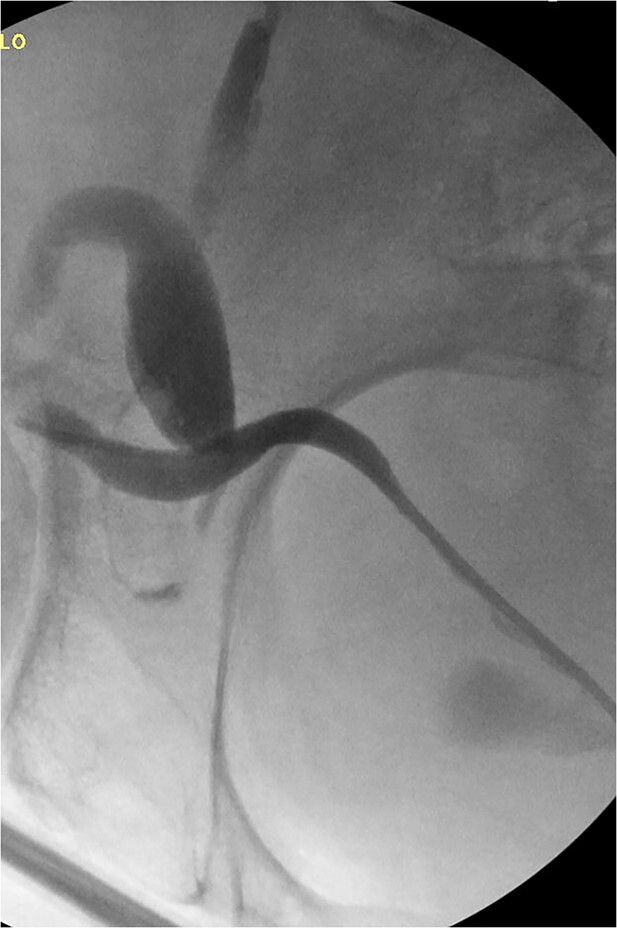
Retrograde pyelogram following hernia repair with tortuous path of the ureter.

The patient was taken to the operative suite where he underwent cystoscopy, retrograde pyelogram and temporary ureteral stent placement (Fig. 2). Right inguinal exploration was performed, and the patient was found to have a large direct hernia containing abundant adipose tissue. The ureter and cord structures were identified. Adipose tissue was amputated with an energy device. The ureter was reduced into the retroperitoneum. The hernia was then repaired with modified Lichtenstein technique using macroporous polypropylene mesh. Retrograde pyelogram was repeated at completion of the hernia repair following stent removal. A serpiginous course of the ureter was noted without obstruction (Fig. 3). He was observed overnight and discharged home the following day. Post-operative course was uneventful.

The patient was allowed to fully recover over the next 4 months. He then returned to the operating suite and a nearly identical procedure was performed on the left side. His post-operative course was uneventful. At a 6-month follow-up, the patient exhibited no recurrence of his hernias, markedly decreased size of the scrotum and significant improvement in urinary soiling due to retraction of the phallus.

CT abdomen performed >5 years after repair revealed intact bilateral inguinal hernia repair and no evidence of hydronephrosis.

## DISCUSSION

Bilateral ureteroinguinal hernia has rarely been described in the literature and there are not clear guidelines for best management. There are two variations of ureteroinguinal hernias, paraperitoneal and extraperitoneal. Paraperitoneal comprises 80% and has an indirect sac that pulls on the ureter. Extraperitoneal occurs without a peritoneal sac, and the ureter moves with the retroperitoneal fat into the scrotum and is thought to be a congenital defect in which the Wolffian duct fails to separate from the ureteric bud [10].

Due to the high volume of inguinal hernia surgery and low incidence of herniated genitourinary organs, it is difficult to recommend preoperative imaging for all patients. Performing a quality preoperative history may be beneficial inquiring about two-stage micturition, hematuria, acute urinary obstruction or ipsilateral flank pain. Patients with suspicious history may warrant preoperative imaging with ultrasound or CT scan [7]. Interestingly, Allam et al. reported in a case series that anterior displacement of the ureter by >1 cm anterior to the psoas at the L4 level on CT was associated with inguinoscrotal herniation of the ureter.

It has been consistently reported that ureteroinguinal hernia is associated with large cord lipomas and sliding fat, especially in obese patients in their 50s and 60s. It is important to consider this possibility to limit iatrogenic ureteral injury.

In patients who have ureteroinguinal hernia identified pre- and post-operatively management options that have been reported include anterior repair with reduction into the retroperitoneum, percutaneous nephrostomy and stenting, retrograde stenting, nephrectomy, exploratory laparotomy and resection of redundant ureter with re-implantation [8, 11]. There is one reported case of addressing ureteroinguinal hernia with a laparoscopic approach [12].

In our patient, we elected to perform repair in a staged fashion to ensure the patient did not develop obstruction prior to proceeding with contralateral repair. Given the large size of the hernias, risk of ureter injury and the decision to perform a staged approach, we chose to perform open repair rather than minimally invasive. Retrograde stenting was beneficial to facilitate identification of ureters and decrease the risk of injury. Retrograde pyelogram after completion of repair confirmed no obstruction prior to leaving the operating suite. This approach resulted in a successful outcome with a greater than 5-year follow-up.
